# Associations between Lifetime Traumatic Events and Subsequent Chronic Physical Conditions: A Cross-National, Cross-Sectional Study

**DOI:** 10.1371/journal.pone.0080573

**Published:** 2013-11-19

**Authors:** Kate M. Scott, Karestan C. Koenen, Sergio Aguilar-Gaxiola, Jordi Alonso, Matthias C. Angermeyer, Corina Benjet, Ronny Bruffaerts, Jose Miguel Caldas-de-Almeida, Giovanni de Girolamo, Silvia Florescu, Noboru Iwata, Daphna Levinson, Carmen C. W. Lim, Sam Murphy, Johan Ormel, Jose Posada-Villa, Ronald C. Kessler

**Affiliations:** 1 Department of Psychological Medicine, University of Otago, Dunedin, New Zealand; 2 Columbia Mailman School of Public Health, New York, New York, United States of America; 3 Center for Reducing Health Disparities (CRHD), Community Engagement Program of the Clinical Translational Science Center (CTSC), University of California Davis, School of Medicine, Sacramento, California, United States of America; 4 Health Services Research Unit, Institut Municipal d Investigacio Medica (IMIM-Hospital del Mar), and CIBER en Epidemiologıa y Salud Publica (CIBERESP), Barcelona, Spain; 5 Center for Public Mental Health, Gösing/Wagram, Austria; 6 Department of Epidemiologic and Psychosocial Research, National Institute of Psychiatry Ramón de la Fuente, Mexico City, Mexico; 7 Universitair Psychiatrisch Centrum - Katholieke Universiteit Leuven (UPC-KUL), Leuven, Belgium; 8 Chronic Diseases Research Center (CEDOC) and Department of Mental Health, Faculdade de Ciencias Medicas, Universidade Nova de Lisboa, Lisbon, Portugal; 9 IRCCS Centro S. Giovanni di Dio Fatebenefratelli, Brescia, Italy; 10 National School of Public Health, Management and Professional Development, Bucharest, Romania; 11 Department of Clinical Psychology, Faculty of Psychological Sciences, Hiroshima International University, Hiroshima, Japan; 12 Mental Health Services, Ministry of Health, Jerusalem, Israel; 13 Department of Psychological Medicine, Otago University, Dunedin, New Zealand; 14 Psychology Research Institute, School of Psychology, University of Ulster, Londonderry, Northern Ireland; 15 University of Groningen, University Medical Center Groningen, Groningen, The Netherlands; 16 Colegio Mayor de Cundinamarca University, Bogota, DC, Colombia; 17 Department of Health Care Policy, Harvard Medical School, Boston, Massachusetts, United States of America; Cardiff University, United Kingdom

## Abstract

**Background:**

Associations between lifetime traumatic event (LTE) exposures and subsequent physical ill-health are well established but it has remained unclear whether these are explained by PTSD or other mental disorders. This study examined this question and investigated whether associations varied by type and number of LTEs, across physical condition outcomes, or across countries.

**Methods:**

Cross-sectional, face-to-face household surveys of adults (18+) were conducted in 14 countries (n = 38, 051). The Composite International Diagnostic Interview assessed lifetime LTEs and DSM-IV mental disorders. Chronic physical conditions were ascertained by self-report of physician's diagnosis and year of diagnosis or onset. Survival analyses estimated associations between the number and type of LTEs with the subsequent onset of 11 physical conditions, with and without adjustment for mental disorders.

**Findings:**

A dose-response association was found between increasing number of LTEs and odds of any physical condition onset (OR 1.5 [95% CI: 1.4–1.5] for 1 LTE; 2.1 [2.0–2.3] for 5+ LTEs), independent of all mental disorders. Associations did not vary greatly by type of LTE (except for combat and other war experience), nor across countries. A history of 1 LTE was associated with 7/11 of the physical conditions (ORs 1.3 [1.2–1.5] to 1.7 [1.4–2.0]) and a history of 5+ LTEs was associated with 9/11 physical conditions (ORs 1.8 [1.3–2.4] to 3.6 [2.0–6.5]), the exceptions being cancer and stroke.

**Conclusions:**

Traumatic events are associated with adverse downstream effects on physical health, independent of PTSD and other mental disorders. Although the associations are modest they have public health implications due to the high prevalence of traumatic events and the range of common physical conditions affected. The effects of traumatic stress are a concern for all medical professionals and researchers, not just mental health specialists.

## Introduction

A mounting body of research has documented associations between traumatic event exposures and poor physical health. Up until recently these associations have been considered to be mediated by posttraumatic stress disorder (PTSD)[1]. This mediation is highly plausible because PTSD and other mental disorders have been repeatedly associated with concurrent and subsequent poor physical health [2]–[6]. Despite this, the role of PTSD remains unclear, because although many studies have found PTSD to mediate associations between traumatic event exposures and physical health [7]–[14], other studies have suggested that trauma exposure has independent associations with physical health problems [15]–[22]. This question of whether traumatic stress is associated with increased risk of physical ill-health independently of PTSD has important implications for the mechanisms linking trauma and physical health and for treatment. At present it is generally assumed that these mechanisms hinge around PTSD and its biobehavioural effects [23], with the implication being that if PTSD is successfully treated, the adverse health impact of trauma will be ameliorated. However if traumatic events were confirmed as independent predictors of poor physical health outcomes, this would provide quite a different perspective on the scale of traumatic stress sequelae, given the much higher prevalence of traumatic event exposure than PTSD.

The discrepant findings on the role of PTSD may be attributable in part to how trauma exposure is measured. Studies finding independent effects of trauma exposure tend to be large, general population studies with measures of the full range of traumas experienced over the lifetime, in contrast to the studies finding an explanatory role for PTSD that have frequently focussed on a single specific trauma exposure like combat, sexual abuse or natural disaster. Taking lifetime trauma history into account is important because the experience of multiple trauma exposures is common [17], [24] and is associated with greater likelihood of and severity of PTSD symptoms [25]–[28], so the effects of PTSD become confounded with multiple traumatic exposures unless they are included in models as separate variables.

A second unresolved issue is whether some types of traumatic events are more ‘toxic’ in their effects than others. This remains unresolved because even among the studies with data on a wide range of traumas, the effect of trauma type has not always been examined [17], [22]. One exception was a recent study by Keyes et al. that found assaultive violence or other threats to physical integrity to be the only trauma types significantly associated with any of the medical conditions, but the study may have underpowered for this investigation [15]. More research with large enough samples is also needed to determine whether the effects of traumatic events vary across physical outcomes.

A third issue of interest is the nature of the relationship between the number of traumatic events and subsequent physical ill-health. Most studies investigating number of traumas have used a single continuous measure that assumes a linear effect [17], [21], [22]. By contrast, the study by Keyes et al.[15] discretized number of traumas into several categories and observed a J-shaped pattern whereby a small number of trauma exposures was associated with decreased odds of four out of the six physical conditions studied (not all associations were significant), but larger numbers of trauma exposures were associated with increased odds of the physical conditions._ENREF_13 This interesting finding is suggestive of a resilience-building effect of minimal (non-zero) traumatic event exposures [29] but it has not been examined in other studies on this topic to our knowledge.

Finally, the vast majority of prior research in this area has come from the US or similar countries. This makes it unclear whether prior findings can be generalized, as the severity and consequences of traumas may vary across countries [1], [12].

This study uses the data from 14 of the World Mental Health (WMH) Surveys to investigate these unresolved issues. The dataset contains retrospective information on lifetime traumatic event (LTE) exposures, mental disorder diagnoses and self-reported physician diagnoses of chronic physical conditions. Timing (year of occurrence/onset/diagnosis) information was also collected allowing survival analyses of the retrospective data to examine predictive associations between LTEs and the subsequent diagnosis or onset of physical conditions, independent of PTSD and other mental disorders. In this analysis our objectives were to examine: (i) whether there are associations between LTEs and physical conditions independent of mental disorders; (ii) whether associations vary by type and number of LTEs; (iii) whether the associations of LTEs with physical health vary by type of physical condition; (iv) whether associations vary across countries.

## Methods

### Samples and Procedures

#### Ethics Statement

All respondents provided written informed consent and procedures for protecting respondents were approved and monitored for compliance by the Institutional Review Boards in each country (see [31] for details).

All WMH surveys with requisite data were included in this analysis (see Table 1 for sample characteristics). A stratified multi-stage clustered area probability sampling strategy was used to select adult respondents (18 years+) in most WMH countries. Most of the surveys were based on nationally representative household (or population register) samples while Colombia and Mexico were based on nationally representative household samples in urbanized areas. The central WMH staff trained bilingual supervisors in each country. The WHO translation protocol was used to translate instruments and training materials. Some surveys were carried out in bilingual form while others were carried out exclusively in the country's official language. Measures taken to ensure data accuracy and cross-national consistency are described in detail elsewhere [30], [31].

**Table 1:** Characteristics of WMH samples and percent of respondents reporting lifetime traumatic events.

Country	Sample Size		Response Rate (%)	% reporting lifetime traumatic events						% with PTSD
	Part 1	Part 2 sub-sample		1	2	3	4	5+	Any	
**Americas**										
Colombia	4426	2381	87.7	21.5	17.1	13.2	9.9	19.0	80.6	1.5
Mexico	5782	2362	76.6	21.8	14.8	12.2	7.2	9.4	65.3	1.2
United States	9282	5692	70.9	22.8	17.7	13.6	8.2	17.1	79.4	5.9
**Asia and South Pacific**										
Japan	4129	1682	55.1	23.8	16.4	7.9	3.9	4.2	56.1	1.2
New Zealand	12790	7312	73.3	25.4	17.2	12.9	7.3	13.2	75.9	5.7
**Europe**										
Belgium	2419	1043	50.6	24.9	18.3	8.6	3.6	6.9	62.3	2.4
Germany	3555	1323	57.8	24.9	17.8	8.1	6.2	4.9	61.9	1.4
Italy	4712	1779	71.3	25.9	14.2	6.6	2.9	2.6	52.3	1.9
The Netherlands	2372	1094	56.4	27.2	14.8	8.7	4.6	6.1	61.4	3.9
Spain	5473	2121	78.6	27.6	10.4	6.8	3.0	1.8	49.5	1.7
Northern Ireland	4340	1986	68.4	21.6	14.1	7.7	6.1	7.8	57.3	8.2
Portugal	3849	2060	57.3	22.6	18.8	9.5	6.0	7.1	63.9	4.7
Romania	2357	2357	70.9	18.7	7.4	4.3	2.6	2.0	35.0	0.7
**Middle East**										
Israel	4859	4859	72.6	26.8	18.8	12.1	6.6	7.5	71.8	1.4
										
**All countries**				24.1	16.0	10.6	6.2	9.6	66.4	3.5
**Total sample size**	70345	38051								
**Weighted average response rate (%)**			68.7							

In most countries, internal subsampling was used to reduce respondent burden and average interview time by dividing the interview into two parts. Part I of the interviews, which assessed core disorders, was completed by all respondents. Part II assessed additional disorders, including PTSD and physical conditions, and was completed by 100% of respondents who met criteria for any Part I disorder and a probability subsample of other Part I respondents. The Part II sample was used in this study. The Part I sample was weighted to adjust for differential probabilities of selection and residual discrepancies between sample and census on sociodemographic and geographic variables. The Part II sample was additionally weighted to adjust for undersampling of Part I respondents without Part I disorders, resulting in an unbiased sample. World Mental Health sampling and weighting are discussed in more detail elsewhere [31].

### Measures

#### Mental disorders

All surveys used the WMH survey version of the WHO Composite International Diagnostic Interview (CIDI 3.0 [30]), a fully structured interview, to assess lifetime history of mental disorders including their age of onset. Disorders were assessed using the definitions and criteria of the DSM-IV. In addition to PTSD (asked about in relation to both a respondent's worst trauma and, in those with more than one traumatic event, a randomly selected trauma), other lifetime DSM-IV/CIDI disorders included in this paper are anxiety disorders (panic disorder, agoraphobia, social phobia, specific phobia, generalized anxiety disorder, obsessive compulsive disorder), mood disorders (major depressive disorder/dysthymic disorder, bipolar disorder), impulse control disorders (intermittent explosive disorder, bulimia, binge eating disorder) and substance use disorders (alcohol or drug abuse/dependence). As described elsewhere [32], blinded SCID clinical reappraisal interviews in several of the WMH countries documented generally good CIDI-SCID concordance, although the CIDI is conservative relative to the SCID for lifetime estimates.

#### Lifetime potentially traumatic events (LTEs)

The CIDI PTSD section began with questions about lifetime occurrence of 27 LTEs (see table). An additional open-ended question was asked about other types of traumatic event. A second open-ended question then asked about qualifying events that respondents did not report because of embarrassment. Wording was as follows: “[s]ometimes people have experiences they don’t want to talk about in interviews. I won’t ask you to describe anything like this, but without telling me what it was, did you ever have a traumatic event that you did not report to me because you did not want to talk about it?” Positive responses to these questions were recorded verbatim and reviewed subsequently by a clinician to confirm PTSD criterion A1 (that is, that the person has been exposed to a traumatic event involving actual or threatened death or injury). Additional questions were asked to determine the age at which they had first occurred and frequency of occurrence.

#### Chronic physical conditions

These were assessed with a checklist based on the US National Health Interview Survey. Respondents were asked whether they ever had a series of symptom-based conditions (e.g., chronic headaches, chronic low back pain) and whether a health professional had ever told them they had a series of medical conditions (e.g., cancer, hypertension). Previous methodological research has shown that such checklists yield reports that have reasonable to good concordance with diagnoses based on medical records [33], [34]. For all conditions reported, respondents were asked how old they were when they were first diagnosed with the condition (for the medical diagnoses) or first experienced the condition (for the symptomatic conditions). This year is referred to herein as the age of onset of these conditions, although it is recognized that the underlying pathology of these conditions usually develops over many years. Eleven conditions were included in this study as outcome variables with case numbers as follows (after exclusion of those people reporting life-threatening illness as the traumatic event): arthritis (n = 4857); chronic back or neck pain (n = 9353); frequent or severe headaches (n = 7898); heart disease (n = 1480); hypertension (n = 5262); asthma (n = 1231); diabetes (n = 1578); peptic ulcer (n = 2333); cancer (n = 477); other chronic lung disease (n = 468); stroke (n = 355). For all but three of these conditions (back/neck pain; headache; ulcer), only adult onset (21 years+) cases were classified as the outcome variables.

### Statistical Analysis

Discrete-time survival models [35] with person-year as the unit of analysis were used to investigate sequential associations between a prior history of LTE (type and number) and the subsequent onset of ‘any physical condition’ as well as specific physical conditions. The discrete-time approach was chosen over the more traditional Cox proportional hazards approach for two reasons: because our information about the timing of the covariates was only available for discrete intervals of time (i.e., years of age); and because the discrete-time approach handles the use of multiple time-varying predictor variables, which is a feature of these models, much more easily than does the Cox modelling approach.

Eleven person-year data sets were created (one for each physical condition) in which each year in the life of each respondent up to and including the age of onset of the physical condition or their age at interview (whichever came first) was treated as a separate observational record, with the year of the onset of the physical condition coded 1 and earlier years coded 0 on a dichotomous outcome variable. Lifetime traumatic events were coded 1 from the year of their reported first occurrence. Only person-years up to the onset of the physical conditions were analyzed so that only traumatic events occurring prior to the physical condition onsets were included in the predictor set. Other covariates were coded as time invariant (e.g., gender, coded for each person year), or time varying (e.g. each specific mental disorder, coded 1 from year of onset). Respondents who reported life threatening illness or injury as their LTE were excluded from analysis. These data were analyzed using logistic regression models with the survival coefficients presented as odds ratios, indicating the relative odds of physical condition onset in a given year for a person with a history of a specific type or number of traumatic events, compared to people without that history. The odds ratios can be interpreted as survival coefficients because of the exclusion from the data array of person-years after the onset of the outcome.

For analyses where ‘any physical condition’ was the outcome variable, these eleven person year datasets were stacked into a single dataset, including a dummy variable that distinguished among the physical conditions. For these analyses, the effects of the predictor variables are forced to be the same across the eleven different physical conditions. Additional models were estimated with specific physical conditions as outcome variables using the relevant person-year dataset. A series of bivariate and multivariate models were developed including the predictor LTEs plus control variables and mental disorders. Models control for person-years, countries, gender, age-cohorts, years of education, and in the multivariate models, other LTEs and mental disorders. These models adjust for the lifetime history of all mental disorders occurring prior to the physical condition onset, not only those mental disorders occurring between the traumatic event and the physical condition onset. Traumatic event *type* models (using 14 categories derived from the original 29 events) and *number* models (number of event types experienced, with separate dummy variables for 1 event type, 2 event types and so on) were developed. More complex models including both type and number of LTEs were also estimated but the number models were the best fitting models (model fitting statistics available on request) so for subsequent analyses we only report the results from the number models. The number models were estimated for both number of individual events experienced (1–28, excluding life-threatening illness) and number of event types experienced (1–14). Results were virtually identical across these two sets of models and only the results from the *number of event types* models are reported here, but to avoid confusion (with the type models), we refer to the predictors in these models as *number of events or number of LTEs*.

As the WMH data are both clustered and weighted, the design-based Taylor series linearization [36] implemented in version 10 of the SAS-callable SUDAAN software system was used to estimate standard errors and evaluate the statistical significance of coefficients.

## Results

### Sample characteristics

The survey characteristics and the % of respondents reporting the occurrence of LTEs are shown in Table 1. Pooling across all surveys, 66.4% reported at least one LTE, ranging from 35% of respondents in Romania to 80.6% in Colombia. The experience of multiple LTEs was the norm: in all but one country (Spain) the majority of respondents who reported at least one LTE experienced more than one. Table 2 lists the 29 individual LTEs asked about and shows their aggregation into the 14 event subtypes used in the analysis.

**Table 2:** The potentially traumatic events assessed in the World Mental Health Surveys.

Event Subtypes	Individual Traumatic Events (29)
**I. Combat Experience**	Combat experience (military or non-military) in a war or sectarian violence (e.g. political, religious, or ethnic conflicts)
	Purposefully injured, tortured or killed someone
**II. Other War Experience**	Relief worker or peacemaker in war zone or region of sectarian violence
	Civilian in war zone
	Civilian in region of sectarian violence
	Dispatched refugee from a war zone or region of sectarian violence
	Witnessed attrocities or carnage
**III. Physically abused as a child**	Beaten up as a child by a caregiver
**IV. Physically assaulted or threatened**	Kidnappped or held captive
	Beaten up by someone else
	Mugged or threatened with a weapon
**V. Physically assaulted or threatened by spouse or romantic partner**	Beaten up by a spouse or romantic partner
	Stalked
**VI. Sexually assaulted**	Raped
	Sexually assaulted or molested
**VII. Automobile accident**	Life-threatening automobile
**VIII. Other life-threatening accident**	Toxic chemical exposure
	Other life-threatening accident
	Other exposure to a made disaster (e.g. fire explosion at a place of work)
	Accidentally caused injury or death to someone
**IX. Natural disaster**	Natural disaster (e.g., flood, hurricane, earthquake)
**X. Life-threatening illness**	Life threatening illness or injury
**XI. Death**	Unexpected death of a loved one
**XII. Other PLE to a loved one**	Life-threatening illness of a loved one
	Any other trauma experienced by a loved one
**XIII. Witnessed a traumatic injury or death**	Witnessed death/dead body or saw someone seriously hurt
**XIV. Witnessed family violence as a child**	Witnessed repeated physical fights at home as a child
**XV. Other**	Any other objectively qualifying experiences (respondents are asked to describe these experiences)
	Private experiences (respondents are explicitly told in advance that they will not be asked to describe these experiences)

### Associations between type and number of LTEs with the subsequent onset of any physical condition

The bivariate associations shown in Table 3 reveal small to moderate associations between all LTE types (except combat experience) with any physical condition onset (averaging across all types of physical condition). In the next model shown in Table 3, the multivariate type model, the associations between specific LTE types and any physical condition are provided, adjusting for all other types of LTE. As noted above, most people experience multiple LTEs so it is not surprising that adjustment for this attenuates the associations for specific LTEs. Nonetheless all remain modestly associated with increased risk of the outcome with the exceptions of combat and other war experience which become associated with decreased risk of subsequent physical conditions. The test for variation in ORs indicates that these associations differ significantly from one another in magnitude (**χ_15_^2^** = 141.2, p<0.05). The multivariate number model shown in Table 3 reveals a monotonic association between number of LTEs and any subsequent physical condition with ORs ranging from 1.5 to 2.6, with all categories significantly associated with physical ill-health.

**Table 3:** Bivariate and multivariate associations (odds ratios) between type and number of lifetime traumatic events and the subsequent onset of any physical condition.

Any physical condition onset	Bivariate Models^1^		Multivariate Type Model^2^		Multivariate Number Model^3^	
	OR	(95% C.I)	OR	(95% C.I)	OR	(95% C.I)
**I. Lifetime traumatic events: type**						
Combat Experience	1.0	(0.9–1.1)	0.9*	(0.8–1.0)	-	-
Other War Experience	1.1*	(1.0–1.2)	0.9*	(0.9–1.0)	-	-
Physically abused as a child	1.7*	(1.6–1.8)	1.3*	(1.2–1.4)	-	-
Physically assaulted or threatened	1.5*	(1.5–1.6)	1.2*	(1.2–1.3)	-	-
Physically assaulted or threatened by spouse or romantic partner	1.5*	(1.4–1.7)	1.2*	(1.1–1.3)	-	-
Sexually assaulted	1.6*	(1.5–1.7)	1.3*	(1.2–1.4)	-	-
Automobile accident	1.6*	(1.5–1.7)	1.3*	(1.3–1.4)	-	-
Other life-threatening accident	1.5*	(1.4–1.6)	1.2*	(1.1–1.3)	-	-
Natural disaster	1.4*	(1.3–1.5)	1.2*	(1.1–1.3)	-	-
Death	1.4*	(1.3–1.5)	1.2*	(1.2–1.3)	-	-
Other PLE to a loved one	1.4*	(1.3–1.5)	1.1*	(1.1–1.2)	-	-
Witnessed a traumatic injury or death	1.4*	(1.3–1.5)	1.2*	(1.1–1.2)	-	-
Witnessed family violence as a child	1.5*	(1.4–1.6)	1.2*	(1.1–1.3)	-	-
Other	1.3*	(1.3–1.4)	1.1*	(1.0–1.2)	-	-
**Joint effect of all types of events, χ** 2 **_14_**		-		**919.0** *		-
**Difference between types of events, χ** 2 **_13_**		-		**141.2** *		-
**II. Lifetime traumatic events: number**						
1 event	-	-	-	-	1.5*	(1.4–1.6)
2 events	-	-	-	-	1.8*	(1.7–1.9)
3 events	-	-	-	-	2.0*	(1.9–2.1)
4 events	-	-	-	-	2.1*	(2.0–2.3)
5+ events	-	-	-	-	2.6*	(2.3–2.8)
**Joint effect of number of events, χ** 2 **_5_**		-		-		**771.3** *

* Significant at p<0.05 level, 2 sided test

1 Each trauma type was estimated as a predictor of the physical condition onset in a separate discrete time survival model controlling for person-years, age-cohorts, gender, country, education and type of physical condition.

2 The model was estimated with dummies for all trauma types entered simultaneously, including the controls specified above.

3 The model was estimated with dummy predictors for number of trauma types without any information about type of trauma, including the controls specified above.

In further analyses (data not shown but available on request) we examined whether associations between LTE (types and number) and physical condition onset varied by gender or age. We did not find substantive gender variation. In terms of age variation, we observed a consistent pattern of somewhat stronger associations between LTEs and physical condition onset among younger respondents compared to older respondents.

### Association between the number of LTEs and any physical condition, with adjustment for mental disorders

Table 4 shows the effect of progressive adjustment for PTSD (second column), then all mental disorders (third column). The magnitude of associations between number of LTEs and any physical condition changes very little with adjustment for PTSD indicating that these associations are independent of PTSD. An additional analysis (not shown) found no difference in associations after including subthreshold PTSD symptoms. PTSD has an independent association (OR: 1.3) with any physical condition. Once all mental disorders are taken into account however, PTSD is no longer significant and the ORs for number of LTEs are attenuated slightly, mostly noticeably for 5+ events. Several of the mental disorders have small independent associations with the physical condition outcome.

**Table 4:** Multivariate associations (odds ratios) between the number of lifetime traumatic events and the subsequent onset of any physical condition, without and with adjustment for mental disorders.

Any physical condition onset	Multivariate Number Model^1^		Multivariate Number Model (Adjusted for PTSD)^2^		Multivariate Number Model (Adjusted for all mental disorders - type)^3^		
	OR	(95% C.I)	OR	(95% C.I)		OR	(95% C.I)
**I. Lifetime traumatic events: number**							
1 event	1.5*	(1.4–1.6)	1.5*	(1.4–1.6)		1.5*	(1.4–1.5)
2 events	1.8*	(1.7–1.9)	1.8*	(1.7–1.9)		1.7*	(1.6–1.8)
3 events	2.0*	(1.9–2.1)	2.0*	(1.8–2.1)		1.9*	(1.7–2.0)
4 events	2.1*	(2.0–2.3)	2.1*	(1.9–2.3)		2.0*	(1.8–2.1)
5+ events	2.6*	(2.3–2.8)	2.5*	(2.2–2.7)		2.1*	(2.0–2.3)
**Joint effect of number of events, χ** 2 **_5_**		771.3*		709.3*			575.9*
**II. Lifetime mental disorders**							
Major depressive episode/dysthymia	-	-	-	-		1.2*	(1.1–1.2)
Bipolar disorder (broad)	-	-	-	-		1.1	(0.9–1.2)
Panic disorder	-	-	-	-		1.2*	(1.1–1.3)
Generalized anxiety disorder	-	-	-	-		1.1*	(1.0–1.2)
Social phobia	-	-	-	-		1.2*	(1.1–1.3)
Specific phobia	-	-	-	-		1.3*	(1.2–1.4)
Agoraphobia without panic	-	-	-	-		1.1	(0.9–1.2)
Post-traumatic stress disorder	-	-	1.3*	(1.2–1.4)		1.1	(1.0–1.2)
Obsessive compulsive disorder	-	-	-	-		1.3	(1.0–1.6)
Intermittent explosive disorder	-	-	-	-		1.2*	(1.1–1.4)
Binge eating disorder	-	-	-	-		1.2	(1.0–1.5)
Bulimia nervosa	-	-	-	-		1.3*	(1.1–1.6)
Alcohol abuse	-	-	-	-		1.2*	(1.1–1.3)
Alcohol dependence	-	-	-	-		1.0	(0.9–1.2)
Drug abuse	-	-	-	-		1.0	(0.9–1.1)
Drug dependence	-	-	-	-		1.1	(0.9–1.3)

* Significant at p<0.05 level, 2-sided test.

1 The model was estimated with dummy predictors for number of trauma types without any information about type of trauma, controlling for person-years, age-cohorts, gender, country, education and physical condition type.

2 The model was estimated with dummy predictors for number of trauma types without any information about type of trauma and additionally adjusted for PTSD, including the controls specified above.

3 The model was estimated with dummy predictors for number of trauma types without any information about type of trauma and additionally adjusted for all mental disorders, including the controls specified above.

### Associations between the number of LTEs and specific physical conditions, adjusted for all mental disorders

Table 5 shows a monotonic relationship between increasing number of LTEs and risk of most of the specific physical conditions that is independent of mental disorders, but there are exceptions. Those exceptions include high blood pressure and diabetes where at least 1 LTE is associated with the physical condition but there is no increasing risk of the condition with increasing number of LTEs. The other exceptions are cancer and stroke, where there is no significant association with LTEs and the condition. For stroke, although the individual OR for 5+events is significant, the lack of significant chi square value for the joint effect of number of events in this model means this single significant coefficient may not be reliable.

**Table 5:** Multivariate associations (odds ratios) between the number of lifetime traumatic events and the subsequent onset of specific physical conditions, adjusted for mental disorders^1^.

Type of Physical Condition	1 event		2 events		3 events		4 events		5+ events		Joint effect of number of events, χ^2^_5_
	OR	(95% C.I)	OR	(95% C.I)	OR	(95% C.I)	OR	(95% C.I)	OR	(95% C.I)	
Arthritis	1.3*	(1.2–1.5)	1.4*	(1.2–1.6)	1.9*	(1.6–2.2)	1.8*	(1.5–2.1)	2.3*	(1.9–2.6)	156.3*
Back and Neck Pain	1.7*	(1.6–1.9)	2.3*	(2.1–2.5)	2.4*	(2.1–2.7)	2.4*	(2.1–2.8)	3.0*	(2.6–3.5)	526.8*
Frequent or Severe Headaches	1.6*	(1.5–1.7)	2.1*	(1.9–2.3)	2.3*	(2.0–2.6)	2.6*	(2.3–3.1)	2.5*	(2.1–2.8)	359.7*
Heart Disease	1.3*	(1.1–1.6)	1.4*	(1.1–1.8)	1.3	(1.0–1.7)	1.5*	(1.1–2.1)	2.2*	(1.6–3.1)	24.7*
High Blood Pressure	1.4*	(1.2–1.5)	1.3*	(1.1–1.4)	1.4*	(1.2–1.6)	1.4*	(1.2–1.7)	1.3*	(1.0–1.5)	45.8*
Asthma	1.3	(1.0–1.6)	1.4*	(1.1–1.8)	1.4*	(1.1–1.9)	1.8*	(1.2–2.7)	1.8*	(1.3–2.4)	15.4*
Diabetes	1.7*	(1.4–2.0)	1.5*	(1.2–1.9)	1.8*	(1.4–2.4)	1.9*	(1.4–2.5)	1.8*	(1.3–2.5)	40.5*
Peptic Ulcer	1.6*	(1.4–1.9)	2.1*	(1.7–2.5)	2.6*	(2.0–3.3)	2.5*	(1.9–3.3)	2.9*	(2.2–3.8)	94.0*
Cancer	1.1	(0.8–1.6)	1.2	(0.8–1.9)	1.2	(0.7–1.9)	0.8	(0.5–1.3)	1.3	(0.8–2.1)	3.7
Other Chronic Lung Diseases	1.1	(0.8–1.6)	2.3*	(1.5–3.4)	2.8*	(1.7–4.4)	3.0*	(1.8–5.0)	3.6*	(2.0–6.5)	42.8*
Stroke	1.3	(0.8–1.9)	1.6	(1.0–2.6)	1.1	(0.6–1.9)	1.2	(0.5–2.7)	2.4*	(1.3–4.6)	10.0

* Significant at p<0.05 level, 2-sided test.

1 Each row represents a separate multivariate model and each model was estimated with dummy predictors for number of trauma types without any information about type of trauma, controlling for person-years, country, age-cohorts, gender, mental disorders and education.

### Country-specific associations, adjusted for all mental disorders

The final table (6) depicts associations between number of LTEs and any physical condition independent of mental disorders, in each country. Although not all individual categories of LTE number are significant in all countries, all countries with the exception of Northern Ireland show significant associations between number of LTEs and physical ill-health. In all countries (apart from Northern Ireland) there is some indication of increasing strength of association with increasing number of LTEs.

**Table 6:** Multivariate associations (country-specific odds ratios) between the number of lifetime traumatic events and the subsequent onset of any physical condition, adjusted for mental disorders^1^.

Any physical condition onset	1 event		2 events		3 events		4 events		5 events		Joint effect of number of events, χ^2^_5_
	OR	(95% C.I)	OR	(95% C.I)	OR	(95% C.I)	OR	(95% C.I)	OR	(95% C.I)	
Colombia	1.4*	(1.1–1.7)	1.9*	(1.6–2.3)	2.2*	(1.7–2.8)	2.4*	(1.8–3.3)	1.9*	(1.4–2.6)	73.4*
Mexico	1.3	(1.0–1.7)	2.5*	(1.9–3.3)	2.4*	(1.7–3.4)	3.2*	(2.1–4.9)	3.5*	(2.5–5.0)	85.5*
Germany	1.6*	(1.3–2.0)	1.9*	(1.3–2.7)	1.6*	(1.1–2.5)	1.3	(0.9–1.9)	3.1*	(1.8-5.5)	33.2*
Israel	1.4*	(1.2–1.5)	1.4*	(1.3–1.6)	1.5*	(1.3–1.7)	1.7*	(1.4–2.1)	2.0*	(1.6–2.5)	68.5*
Italy	1.5*	(1.2–1.8)	1.8*	(1.4–.2.3)	1.7*	(1.2–2.5)	1.6*	(1.0–2.6)	2.3*	(1.5–3.4)	46.6*
Japan	1.5*	(1.1–1.9)	1.7*	(1.3–2.2)	1.8*	(1.4–2.4)	2.1*	(1.4–3.0)	1.9*	(1.1–3.1)	35.8*
Belgium	1.7*	(1.3–2.3)	1.7*	(1.2–2.5)	2.6*	(1.6–4.2)	2.7*	(1.7–4.3)	2.6*	(1.4–4.6)	64.7*
Netherlands	1.4*	(1.0–1.8)	1.4	(0.9–2.1)	2.0*	(1.3–3.0)	1.0	(0.6–1.6)	2.3*	(1.3–4.0)	20.6*
New Zealand	1.5*	(1.3–1.7)	1.8*	(1.6–2.2)	1.8*	(1.5–2.1)	2.1*	(1.8–2.6)	2.4*	(1.9–2.9)	87.6*
Northern Ireland	1.3*	(1.0–1.5)	1.3	(0.9–1.7)	1.3	(0.9–1.9)	1.2	(0.8–1.8)	1.2	(0.8–1.9)	6.5
Portugal	1.6*	(1.3–1.9)	1.4*	(1.1–1.7)	1.8*	(1.3–2.7)	1.8*	(1.2–2.8)	2.0*	(1.2–3.2)	33.0*
Romania	1.4*	(1.2–1.7)	1.5*	(1.0–2.1)	1.9*	(1.3–2.9)	1.8*	(1.1–2.8)	1.4	(0.7–2.8)	45.3*
Spain	1.4*	(1.2–1.7)	1.9*	(1.5–2.5)	2.2*	(1.6–3.2)	1.1	(0.6–1.9)	1.1	(0.7–1.9)	50.1*
US	1.5*	(1.3–1.8)	1.9*	(1.6–2.1)	2.2*	(1.9–2.6)	2.2*	(1.8–2.8)	2.4*	(2.0–2.9)	185.6*

* Significant at p<0.05 level, 2-sided test.

1 Each row represents a separate multivariate model for each country and each model was estimated with dummy predictors for number of trauma types without any information about type of trauma, controlling for person-years, age-cohorts, gender, mental disorders, physical condition types and education.

## Discussion

In this cross-national study we sought to determine whether there were independent associations between lifetime traumatic event (LTE) exposures and the subsequent onset of a range of chronic physical conditions, after taking lifetime mental disorders into account. We found these associations between most types of traumatic event exposure and 9 out of 11 of the chronic physical conditions studied (cancer and stroke were the exceptions). Associations were independent of PTSD, although the lifetime experience of all mental disorders explained a small portion of the effects of multiple traumas. Several mental disorders had additive effects, but PTSD did not, once lifetime mental disorder history was taken into account. There was a dose-response association between number of LTEs experienced and likelihood of physical condition onset. Associations showed a high level of consistency across countries, with Northern Ireland being the one exception.

The observational nature of the study precludes conclusions regarding causality. To the extent that LTEs occur randomly, the associations are likely to be causal, but not all such events are random [37]–[39] and the resources available to individuals and communities for coping with traumas are also not randomly distributed. Our adjustment for education may not have fully accounted for possible socioeconomic differences between those with and without LTE exposures. Other limitations include the reliance on retrospective reports of LTEs, which may be susceptible to underreporting or change over time [40], [41], although this is less likely to occur in the case of events that are highly salient and occurring after childhood (as for most of the LTEs in this study). With regard to childhood LTEs, it is reassuring to note that a recent study found little difference in associations between prospectively versus retrospectively ascertained maltreatment and subsequent psychopathology [42].

It is also a considerable limitation that the medical conditions were assessed on the basis of self-report of diagnoses rather than independent verification by a medical practitioner. This limitation is mitigated somewhat by the generally good agreement between self-report of medical diagnoses and physician or medical record confirmation of those diagnoses[33] [34], and by the fact that age of onset or diagnosis of medical conditions has been found to be fairly accurate and reliable [43]. These data limitations mean that misclassifications of either predictors (LTEs) or outcomes (physical conditions) are likely to have occurred to some extent in this study. To the extent that these misclassifications are independent of each other (non-differential) they will have biased associations downwards, towards the null. It is also possible, though, that the associations we report could be biased upwards, for example if the LTEs with more severe consequences are more likely to be recalled or reported. It has also been suggested that there could be over-reporting of traumatic events among people with chronic physical conditions in an attempt to make meaning [44] of their disease. However, two aspects of the current results are inconsistent with this latter suggestion. First, the key result in this paper is of associations between traumatic events and physical conditions among those without mental disorder history, and it is those *with* mental disorders (depression specifically) who have shown bias in reporting of past trauma or negative life events [45], [46]. Second, there is a very strong perception among the lay public that stress contributes to cancer [47], and yet we observed no association between traumatic events and cancer.

One final source of bias in WMH survey samples that is important to note is that respondents may have better mental or physical health than non-respondents or may be biased due to differential selection out of the population through early mortality. We comment below where we think this survival bias may have been influential in our results.

This study also has significant strengths: it is based on a large, general population sample from multiple countries making the results widely generalizable; it uses consistently applied diagnostic measures of DSM-IV mental disorders; it contains lifetime history of traumatic event exposures; and it includes as predictors in the statistical models only those LTEs and mental disorders occurring prior to the onset/diagnosis of the physical condition. These strengths enable this study to document that traumatic event exposures are associated with subsequent physical ill-health even in the absence of PTSD (or any other mental disorders). This finding is consistent with the results of other recent studies with general population samples and measurement of lifetime trauma history [15], [17], [20]–[22]. This consistency, together with the large scale of this study, indicates that this finding of independent associations of LTEs with health outcomes is reliable. This is an important finding because it suggests that the effects of LTEs among those *without* mental disorders (who comprise the majority of those exposed to LTEs) reverberate for longer periods of time, and are more substantial, than has previously been appreciated. Although the associations are generally small in magnitude, this belies their public health significance which stems from the high prevalence of LTEs, the common experience of multiple traumas, the dose-response nature of the associations, and the range and prevalence of physical health conditions affected.

Behavioural pathways may be important mediators of these associations between LTEs and physical health. In reviews, smoking behaviour [48], problematic alcohol consumption [49], and sleep problems [50] have all been found to increase after traumatic event exposure, even among those without PTSD (although more so among those with PTSD). It is not clear how long these changes in health related behaviour last in those without PTSD or other mental disorders, but given the addictive nature of some poor health habits, it may be long enough to influence physical health, particularly in those with multiple event exposures which would prolong the periods of unhealthy behaviours. Non-behavioural biological pathways may also be involved. D'Andrea and et al.[51] review studies suggesting that those with trauma history but without PTSD show abnormalities in cortisol response, cardiovascular arousal and neurocognitive structure and function that are intermediate in degree between those with PTSD and those without trauma exposure. Because research on the consequences of traumatic stress has understandably focused on those with PTSD, it is not well documented how long lasting these biological or behavioural changes in those without PTSD may be. A further explanation that has been put forward to explain associations between trauma and physical ill-health among those without psychiatric distress is the possibility that some trauma-exposed individuals use coping mechanisms that involve some form of emotional inhibition or suppression, and that these coping styles have negative physical consequences [51]. It remains to be clarified whether such coping styles would be prevalent enough in the general population to help explain the results we present here. Further research on the mechanisms linking trauma and health among those without diagnosed mental disorders is clearly warranted.

This is one of the few studies to examine whether there was a difference in the effects of type of LTE after taking all event exposures into account. We found little difference in their relative effects, with the notable exception of combat experience and other war experience which were associated with reduced odds of subsequent physical ill-health. This finding may seem at odds with the many studies documenting later physical poor health in combat veterans [3], [52]–[54]. However, many of those studies have either focused on veterans with PTSD (ie, not the effects of combat experience per se), or have lacked a control group of non-veterans. Our finding of reduced risk of chronic physical conditions associated with prior combat experience could reflect a selection effect, whereby combat participants are screened for physical and psychological health (the so-called ‘healthy warrior’ effect [54]). It may also reflect the ongoing medical attention veterans receive in some countries allowing early biomarkers for some chronic diseases to be picked up and preventive measures put in place. Both this association with combat experience and the similar association we have observed for other war experience may also reflect survival biases [54].

We found that LTEs were associated with almost all the physical conditions we studied, with the exception of cancer and stroke. The null finding for cancer is consistent with the lack of strong evidence from prior prospective studies of associations between stress exposures and cancer incidence [55], [56]; although this is a controversial area [56] with conflicting findings [57], [58]. With regard to stroke, although there is some evidence from prior studies of an association between self-reported stress and stroke [59], it appears to be stronger or more consistent for fatal rather than non-fatal stroke [60], [61], which may explain why we did not find an association with stroke in this study. That said, our case numbers were smaller for stroke than for most other physical conditions and we cannot rule this out as a contributing factor to the lack of significant association.

Stress and trauma have longstanding associations with medically unexplained symptoms and somatization [62]–[64]. Somatization has been proposed to vary culturally and to be one explanation for lower rates of mental disorders in Asian and some low income countries [65], [66]. Given this background, it is interesting that we found these trauma-physical health associations to be quite consistent across countries. This may be attributable to the fact that most of the physical condition outcomes we studied were not of the ‘medically unexplained’ type. However, we were only able to investigate 14 countries, many of which are high income countries with somewhat similar cultural heritage, so further cross-national study with a wider variety of countries would be useful.

In summary, in this large cross-national study we found that LTEs were experienced by majority of the population and that the majority of those experiencing LTEs experienced multiple events. Elevated risk of physical ill-health was associated with a single traumatic event exposure and accrued with more events experienced. This risk was not confined to those who developed PTSD or other mental disorders, although those with a history of mental disorders were at highest risk of physical consequences from traumatic stress. A wide range of LTEs were associated with a wide range of health outcomes, independent of mental disorders, in 13/14 countries. From these findings we conclude that although the vast majority of those who experience traumatic events do not develop mental disorders, in this group the downstream effects of these events on physical health may be more substantial and persistent than previously understood. The effects of traumatic stress are therefore a concern for all medical professionals and researchers, not just mental health specialists.

## References

[1] Schnurr PP, Green BL (2004) Understanding relationships among trauma, post-traumatic stress disorder, and health outcomes. Adv Mind Body Med 20: 18–29. 15068106

[2] Boscarino JA (2004) Posttraumatic stress disorder and physical illness: results from clinical and epidemiologic studies. Ann NYAcad Sci 1032: 141–153. 10.1196/annals.1314.011 15677401

[3] Boscarino JA (2008) A prospective study of PTSD and early-age heart disease mortality among Vietnam veterans: implications for surveillance and prevention. Psychosomatic Medicine 70: 668–676. 18596248 10.1097/PSY.0b013e31817bccaf PMC3552245

[4] Kubzansky LD, Koenen KC (2009) Is posttraumatic stress disorder related to development of heart disease? An update. Cleve Clin J Med 76: S60–S65. 10.3949/ccjm.76.s2.12 PMC2746556 19376986

[5] Scott KM, Von Korff M, Angermeyer MC, Benjet C, Bruffaerts R, et al (2011) The association of childhood adversities and early onset mental disorders with adult onset chronic physical conditions Arch Gen Psychiatry. 68: 838–844. 10.1001/archgenpsychiatry.2011.77 PMC3402030 21810647

[6] Qureshi SU, Pyne JM, Magruder KM, Schulz PE, Kunik ME (2009) The link between post-traumatic stress disorder and physical comorbidities: a systematic review. Psychiatr Q 80: 87–97. 19291401 10.1007/s11126-009-9096-4

[7] Weisberg RB, Bruce SE, Machan JT, Kessler RC, Culpepper L, et al (2002) Nonpsychiatric illness among primary care patients with trauma histories and posttraumatic stress disorder. Psychiatr Serv 53: 848–854. 12096168 10.1176/appi.ps.53.7.848

[8] Kimerling R, Clum G, Wolfe J (2000) Relationships among trauma exposure, chronic posttraumatic stress disorder symptoms, and self-reported health in women: replication and extension. J Trauma Stress 13: 115–128. 10761178 10.1023/A:1007729116133

[9] Vedantham K, Brunet A, Boyer R, Weiss DS, Metzler TJ, et al (2001) Posttraumatic stress disorder, trauma exposure, and the current health of Canadian bus drivers. Can J Psychiatry 46: 149–155. 11280084 10.1177/070674370104600206

[10] Lang AJ, Laffaye C, Satz LE, McQuaid JR, Malcarne VL, et al (2006) Relationships among childhood maltreatment, PTSD, and health in female veterans in primary care. Child Abuse Negl 30: 1281–1292. 17116330 10.1016/j.chiabu.2006.06.005

[11] Wagner AW, Wolfe J, Rotnitsky A, Proctor SP, Erickson DJ (2000) An investigation of the impact of posttraumatic stress disorder on physical health. J Trauma Stress 13: 41–55. 10761173 10.1023/A:1007716813407

[12] Norris FH, Slone LB, Baker CK, Murphy AD (2006) Early physical health consequences of disaster exposure and acute disaster-related PTSD. Anxiety Stress Coping 19: 95–110.

[13] Schnurr PP, Spiro AI (1999) Combat exposure, posttraumatic stress disorder symptoms, and health behaviors as predictors of self-reported physical health in older veterans. J Nerv Ment Dis 187: 353–359. 10379722 10.1097/00005053-199906000-00004

[14] Wachen JS, Shipherd JC, Suvak M, Vogt D, King LA, et al (2013) Posttraumatic stress symptomatology as a mediator of the relationship between warzone exposure and physical health symptoms in men and women. J Trauma Stress 26: 319–328. 23695839 10.1002/jts.21818

[15] Keyes KM, McLaughlin KA, Demmer RT, Cerdá M, Koenen KC, et al (2013) Potentially traumatic events and the risk of six physical health conditions in a population-based sample. Depress Anxiety 30: 451–460. 23495094 10.1002/da.22090 PMC4180235

[16] Irish LA, Gabert-Quillen CA, Ciesla JA, Pacella ML, Sledjeski EM, et al (2013) An examination of PTSD symptoms as a mediator of the relationship between trauma history characteristics and physical health following a motor vehicle accident. Depress Anxiety 30: 475–482. 23225518 10.1002/da.22034 PMC4019011

[17] Sledjeski EM, Speisman B, Dierker LC (2008) Does number of lifetime traumas explain the relationship between PTSD and chronic medical conditions? Answers from the National Comorbidity Survey-Replication (NCS-R). J Behav Med 31: 341–349. 18553129 10.1007/s10865-008-9158-3 PMC2659854

[18] Cloitre M, Cohen LR, Edelman RE, Han H (2001) Posttraumatic stress disorder and extent of trauma exposure as correlates of medical problems and perceived health among women with childhood abuse. Women Health 34: 1–17. 10.1300/J013v34n03_01 PMC5918463 11708684

[19] Norman SB, Means-Christensen AJ, Craske MG, Sherbourne CD, Roy-Byrne PP, et al (2006) Associations between psychological trauma and physical illness in primary care. J Trauma Stress 19: 461–470. 16929502 10.1002/jts.20129

[20] Spitzer C, Barnow S, Völzke H, John U, Freyberger HJ, et al (2009) Trauma, posttraumatic stress disorder, and physical illness: findings from the general population. Psychosom Med 71: 1012–1017. 19834051 10.1097/PSY.0b013e3181bc76b5

[21] Pietrzak RH, Goldstein RB, Southwick SM, Grant BF (2012) Physical health conditions associated with posttraumatic stress disorder in U.S. older adults: results from wave 2 of the National Epidemiologic Survey on Alcohol and Related Conditions. J Am Geriatr Soc 60: 296–303. 22283516 10.1111/j.1532-5415.2011.03788.x PMC3288257

[22] Pietrzak RH, Goldstein RB, Southwick SM, Grant BF (2011) Medical comorbidity of full and partial posttraumatic stress disorder in US adults: results from Wave 2 of the National Epidemiologic Survey on Alcohol and Related Conditions. Psychosom Med 73: 697–707. 21949429 10.1097/PSY.0b013e3182303775 PMC3188699

[23] McFarlane AC (2010) The long-term costs of traumatic stress: intertwined physical and psychological consequences. World Psychiatry 9: 3–10. 20148146 10.1002/j.2051-5545.2010.tb00254.x PMC2816923

[24] Kessler RC, Sonnega A, Bromet EJ, Hughes M, Nelson CB (1995) Posttraumatic stress disorder in the National Comorbidity Survey. Arch Gen Psychiatry 52: 1048–1060. 7492257 10.1001/archpsyc.1995.03950240066012

[25] Suliman S, Mkabile SG, Fincham DS, Ahmed R, Stein DJ, et al (2009) Cumulative effect of multiple trauma on symptoms of posttraumatic stress disorder, anxiety, and depression in adolescents. Comp Psychiatry 50: 121–127. 10.1016/j.comppsych.2008.06.006 19216888

[26] Briere J, Kaltman S, Green BL (2008) Accumulated childhood trauma and symptom complexity. J Trauma Stress 21: 223–226. 18404627 10.1002/jts.20317

[27] Green BL, Goodman LA, Krupnick JL, Corcoran CB, Petty RM, et al (2000) Outcomes of single versus multiple trauma exposure in a screening sample. J Trauma Stress 13: 271–286. 10838675 10.1023/A:1007758711939

[28] Carrion VG, Kletter H (2012) Posttraumatic stress disorder: shifting toward a developmental framework. Child Adolesc Psychiatr Clin N Am 21: 573–591. 22800995 10.1016/j.chc.2012.05.004

[29] Seery MD, Alison Holman E, Cohen Silver R (2010) Whatever does not kill us: cumulative lifetime adversity, vulnerability, and resilience. J Pers Soc Psychol 99: 1025. 20939649 10.1037/a0021344

[30] Kessler RC, Ustun B (2004) The World Mental Health (WMH) Survey Initiative version of the World Health Organization (WHO) Composite International Diagnostic Interview (CIDI). Int J Method Psychiatr Res 13: 93–121. 10.1002/mpr.168 PMC6878592 15297906

[31] Kessler RC, Ustun TB, editors (2008) The WHO World Mental Health Surveys: Global Perspectives on the Epidemiology of Mental Disorders. New York: Cambridge University Press.

[32] Haro JM, Arbabzadeh-Bouchez S, Brugha TS, de Girolamo G, Guyer ME, et al (2006) Concordance of the Composite International Diagnostic Interview Version 3.0 (CIDI 3.0) with standardized clinical assessments in the WHO World Mental Health Surveys. Int J Method Psychiatr Res 15: 167–180. 10.1002/mpr.196 PMC6878271 17266013

[33] Kriegsman DM, Penninx BW, Van Eijk JT, Boeke AJ, Deeg DJ (1996) Self-reports and general practitioner information on the presence of chronic diseases in community dwelling elderly. J Clin Epidemiol 49: 1407–1417. 8970491 10.1016/s0895-4356(96)00274-0

[34] Baumeister H, Kriston L, Bengel J, Harter M (2010) High agreement of self-report and physician-diagnosed somatic conditions yields limited bias in examining mental-physical comorbidity. J Clin Epidemiol 63: 558–565. 19959329 10.1016/j.jclinepi.2009.08.009

[35] Singer JD, Willett JB (1993) It’s about time: using discrete-time survival analysis to study duration and the timing of events. J Educ Stat 18: 155–195.

[36] Shah BV (1998) Linearization methods of variance estimation. In: Armitage P, Colton T, editors. Encyclopedia of Biostatistics. Chichester: John Wiley and Sons. pp. 2276–2279.

[37] Ormel J, Neeleman J (2000) Towards a dynamic stress-vulnerability model of depression. Where Inner and Outer Worlds Meet: Psychological Research in the Tradition of George W Brown. Florence, KY, USA Routledge pp. 151–169.

[38] Tolin DF, Foa EB (2006) Sex differences in trauma and posttraumatic stress disorder: a quantitative review of 25 years of research. Psychol Bull 132: 959. 17073529 10.1037/0033-2909.132.6.959

[39] Breslau N, Davis GC, Andreski P (1995) Risk factors for PTSD-related traumatic events: a prospective analysis. Am J Psychiatry 529–535. 10.1176/ajp.152.4.529 7694900

[40] Fergusson DM, Horwood LJ, Woodward LJ (2000) The stability of child abuse reports: A longitudinal study of young adults. Psychol Med 30: 529–544. 10883709 10.1017/s0033291799002111

[41] Widom CS, Morris S (1997) Accuracy of adult recollections of childhood victimization: Part 2. childhood sexual abuse. Psychol Assess 9: 34–46.

[42] Scott KM, McLaughlin KA, Smith DA, Ellis PM (2012) Childhood maltreatment and DSM-IV adult mental disorders: comparison of prospective and retrospective findings. Brit J Psychiatry 200: 469–475. 22661679 10.1192/bjp.bp.111.103267 PMC3365274

[43] Toren K, Palmqvist M, Lowhagen O, Balder B, Tunsater A (2006) Self-reported asthma was biased in relation to disease severity while reported year of asthma onset was accurate. J Clin Epidemiol 59: 90–93. 16360566 10.1016/j.jclinepi.2005.03.019

[44] Park CL, Edmondson D, Fenster JR, Blank TO (2008) Meaning making and psychological adjustment following cancer: the mediating roles of growth, life meaning, and restored just-world beliefs. J Consult Clin Psychol 76: 863. 18837603 10.1037/a0013348

[45] Williams JMG, Watts FN, MacLeod C, Mathews A, editors (1997) Cognitive Psychology and Emotional Disorders. Second ed. Chichester: John Wiley and Sons.

[46] Blaney PH (1986) Affect and memory: a review. Psychol Bull 99: 229–246. 3515383

[47] Stewart D, Cheung A, Duff S, Wong F, McQuestion M, et al (2001) Attributions of cause and recurrence in long-term breast cancer survivors. Psychooncology 10: 179–183. 11268144 10.1002/pon.497

[48] Feldner MT, Babson KA, Zvolensky MJ (2007) Smoking, traumatic event exposure, and post-traumatic stress: A critical review of the empirical literature. Clin Psychol Rev 27: 14–45. 17034916 10.1016/j.cpr.2006.08.004 PMC2575106

[49] Stewart SH (1996) Alcohol abuse in individuals exposed to trauma: A critical review. Psychol Bull 120: 83–112. 8711018 10.1037/0033-2909.120.1.83

[50] Babson KA, Feldner MT (2010) Temporal relations between sleep problems and both traumatic event exposure and PTSD: A critical review of the empirical literature. J Anxiety Dis 24: 1–15. 10.1016/j.janxdis.2009.08.002 PMC2795058 19716676

[51] D'Andrea W, Sharma R, Zelechoski AD, Spinazzola J (2011) Physical health problems after single trauma exposure: When stress takes root in the body. J Am Psychiatr Nurses Assoc 17: 378–392. 22142975 10.1177/1078390311425187

[52] McAndrew LM, Elizabeth D, Lu S-E, Abbi B, Yan GW, et al (2013) What pre-deployment and early post-deployment factors predict health function after combat deployment?: a prospective longitudinal study of Operation Enduring Freedom (OEF)/Operation Iraqi Freedom (OIF) soldiers. Health Qual Life Outcomes 11: 73. 23631419 10.1186/1477-7525-11-73 PMC3704953

[53] Vaccarino V, Goldberg J, Rooks C, Shah AJ, Veledar E, et al.. (2013) Posttraumatic stress disorder and incidence of coronary heart disease: A twin study. J American Coll Cardiol. 10.1016/j.jacc.2013.04.085 PMC3823367 23810885

[54] Tansey CM, Raina P, Wolfson C (2013) Veterans' physical health. Epidemiol Rev 35: 66–74. 23243049 10.1093/epirev/mxs005

[55] Cohen S, Janicki-Deverts D, Miller GE (2007) Psychological stress and disease. JAMA 298: 1685–1687. 17925521 10.1001/jama.298.14.1685

[56] Johansen C, Coyne JC, Sanderman R, Dalton SO (2010) Re: Cancer incidence in Israeli Jewish survivors of World War II. J Natl Cancer Inst 102: 991–992. 20516398 10.1093/jnci/djq212

[57] Chida Y, Hamer M, Wardle J, Steptoe A (2008) Do stress-related psychosocial factors contribute to cancer incidence and survival? Nat Clin Pract Oncol 5: 466–475. 18493231 10.1038/ncponc1134

[58] Antonova L, Aronson K, Mueller CR (2011) Stress and breast cancer: from epidemiology to molecular biology. Breast Cancer Res 13: 208. 21575279 10.1186/bcr2836 PMC3219182

[59] Harmsen P, Lappas G, Rosengren A, Wilhelmsen L (2006) Long-term risk factors for stroke twenty-eight years of follow-up of 7457 middle-aged men in Göteborg, Sweden. Stroke 37: 1663–1667. 16728686 10.1161/01.STR.0000226604.10877.fc

[60] Truelsen T, Nielsen N, Boysen G, Grønbæk M (2003) Self-reported stress and risk of stroke: the Copenhagen City Heart Study. Stroke 34: 856–862. 12637696 10.1161/01.STR.0000062345.80774.40

[61] Iso H, Date C, Yamamoto A, Toyoshima H, Tanabe N, et al (2002) Perceived mental stress and mortality from cardiovascular disease among Japanese men and women: The Japan Collaborative Cohort Study for Evaluation of Cancer Risk sponsored by Monbusho (JACC Study). Circulation 106: 1229–1236. 12208798 10.1161/01.cir.0000028145.58654.41

[62] Katon W, Sullivan M, Walker E (2001) Medical symptoms without identified pathology: relationship to psychiatric disorders, childhood and adult trauma, and personality traits. Ann Intern Med 134: 917–925. 11346329 10.7326/0003-4819-134-9_part_2-200105011-00017

[63] Imbierowicz K, Egle UT (2003) Childhood adversities in patients with fibromyalgia and somatoform pain disorder. Eur J Pain 7: 113–119. 12600792 10.1016/S1090-3801(02)00072-1

[64] Spitzer C, Barnow S, Gau K, Freyberger HJ, Grabe HJ (2008) Childhood maltreatment in patients with somatization disorder. Aust NZ J Psychiatry 42: 335–341. 10.1080/00048670701881538 18330776

[65] Shen Y-C, Zhang M-Y, Huang Y-Q, He Y-L, Liu Z-R, et al (2006) Twelve-month prevalence, severity, and unmet need for treatment of mental disorders in metropolitan China. Psychol Med 36: 257–268. 16332281 10.1017/S0033291705006367

[66] Kleinman A (2004) Culture and depression. N Engl J Med 351: 951–952. 15342799 10.1056/NEJMp048078

